# Neuropsychiatric lupus in children

**DOI:** 10.1186/1546-0096-9-S1-P264

**Published:** 2011-09-14

**Authors:** P Costa Reis, S Nativ, J Isgro, C Yildirim-Toruner, L Imundo, A Eichenfield

**Affiliations:** 1 Department of Pediatrics, Santa Maria Hospital, Lisbon, Portugal; 2 Division of Rheumatology, Morgan Stanley Children’s Hospital of New York-Presbyterian, Columbia University Medical Center (MSCHONY/CUMC), New York, USA

## Background

Neuropsychiatric manifestations (NPSLE) are common in children with lupus, and are reported to be associated with worse prognosis.

## Aim

To describe the clinical characteristics of children with NPSLE, including co-morbidities, damage, and mortality.

## Methods

Retrospective chart review of the clinical, laboratory, and radiographic features of SLE patients diagnosed before the age of 18 and followed at MSCHONY/CUMC in 2007-2009. Disease activity (SLEDAI) at diagnosis, occurrence of major infections, and Systemic Lupus International Collaborating Clinics Damage Index (SLICC) were evaluated.

NPSLE was defined according to American College of Rheumatology criteria.

SPSS 17.0 was used for statistical analysis.

## Results

There were 120 children with SLE followed during the study period: 93 female (77.5%, M:F 3.4:1); 61 Hispanic (50.8%) and 34 African-American (28.3%).

NPSLE affected 15 children (12.5%). Cognitive dysfunction was the most common manifestation, followed by significant headache, seizures, psychosis, and focal neurologic signs. NPSLE was diagnosed in the first year in 10/15 patients (67%). Abnormalities of brain magnetic resonance imaging were encountered in 10 patients (67%).

Children with NPSLE had a significantly greater number of co-morbidities (end-stage renal disease, thrombocytopenia, and pancreatitis) than unaffected children with SLE (p<0.05). NPSLE was associated with a higher SLEDAI at diagnosis and the presence of anti-Smith antibodies (p<0.05).

All patients received high-dose corticosteroid and cyclophosphamide. NPSLE was associated with the occurrence of major infections, as well as a higher SLICC damage index, and mortality (p<0.05).

## Conclusions

In this cohort NPSLE was associated with co-morbidities, major infections, and higher damage scores.

